# Characterizing telemedicine use in clinical immunology and allergy in Canada before the COVID-19 pandemic

**DOI:** 10.1186/s13223-021-00635-1

**Published:** 2021-12-13

**Authors:** Erika Yue Lee, Christine Song, Peter Vadas, Matthew Morgan, Stephen Betschel

**Affiliations:** 1 Faculty of Medicine, University of Toronto, Toronto, ON Canada; 2 Division of Clinical Immunology and Allergy, St. Michael’s Hospital, 30 Bond Street, Toronto, ON M5B 1W8 Canada; 3 Division of General Internal Medicine, Mount Sinai Hospital, Toronto, ON Canada

## Abstract

**Rationale:**

There exists a geographic barrier to access CIA care for patients who live in rural communities; telemedicine may bridge this gap in care. Herein we characterized the use of telemedicine in CIA at a population-based level and single centre.

**Methods:**

Before the COVID-19 pandemic, telemedicine care was provided via the Ontario Telemedicine Network (OTN) in Ontario, Canada. Descriptive data were collected from the OTN administrative database and from electronic medical records at a single academic centre during 2014 to 2019. The potential distance travelled and time saved by telemedicine visits were calculated using postal codes.

**Results:**

A total of 1298 telemedicine visits was conducted over OTN, with an average of 216 visits per year. Only 11% of the allergists/immunologists used telemedicine to provide care before the COVID-19 pandemic. In the single centre that provided the majority of the telemedicine care, 66% patients were female and the overall mean age was 46. The most common diagnosis was immunodeficiency (40%), followed by asthma (13%) and urticaria (11%). Most patients required at least one follow-up via telemedicine. The average potential two-way distance travelled per visit was 718 km and the average potential time travelled in total was 6.6 h.

**Conclusion:**

Telemedicine was not widely used by allergists/immunologists in Ontario, Canada before the COVID-19 pandemic. It could offer a unique opportunity to connect patients who live in remote communities and allergists/immunologists who practice in urban centres in Canada. Independent of the current pandemic, our study further highlights the need for more physicians to adopt and continue telemedicine use as well as for healthcare agencies to support its use as a strategic priority once the pandemic is over.

## Introduction

Synchronous telemedicine refers to the delivery of care using an interactive audio–video communication system, where physicians provide care to patients in real-time [1, 2]. Even before the COVID-19 pandemic, its use in Clinical Immunology and Allergy (CIA) has been increasing in the US, particularly for adverse drug reactions and immunodeficiency [3]. Although telemedicine has been available in Canada to provide care to patients who live in remote areas and lack access to allergists/immunologists, the pattern of its use has not been evaluated.

**Fig. 1:**
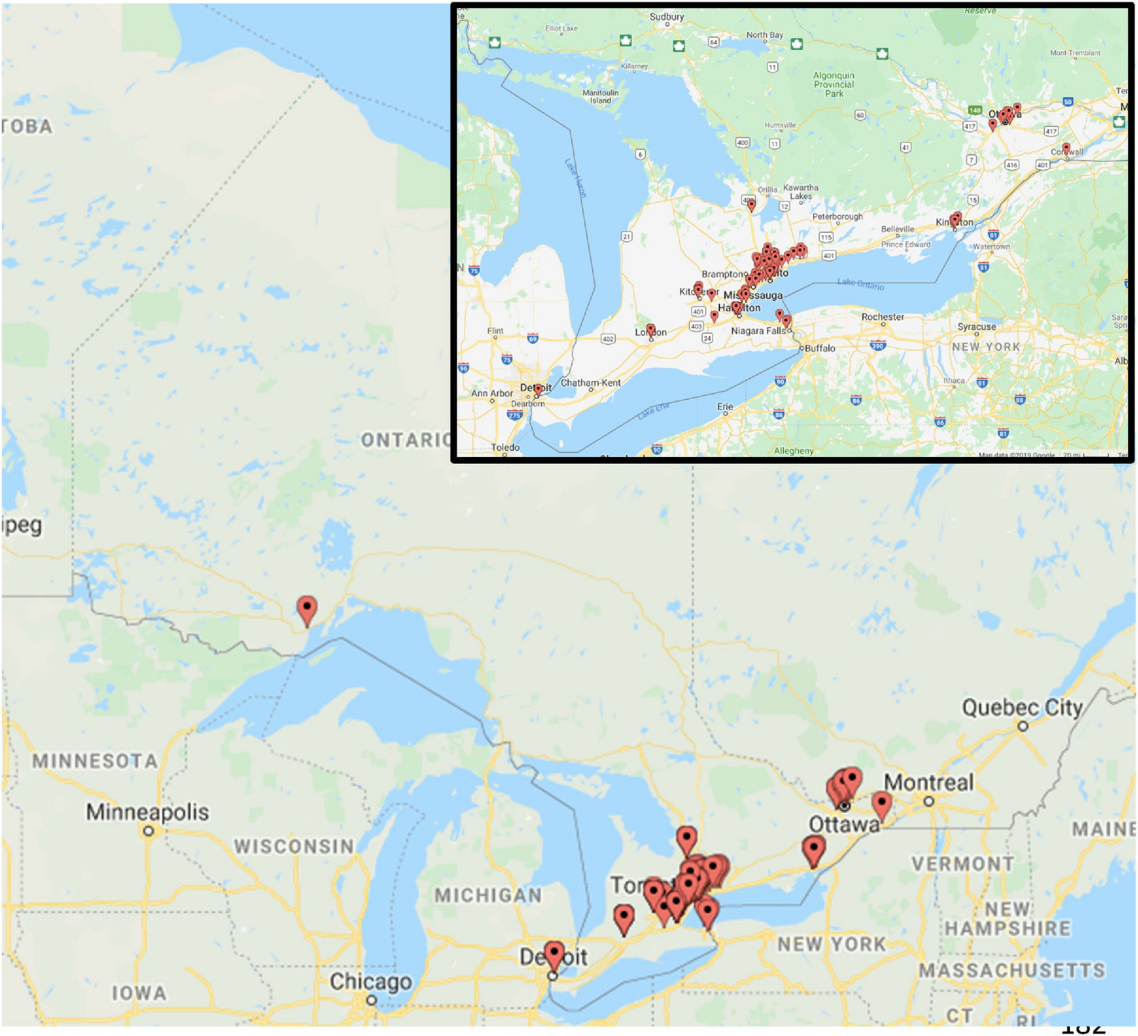
The geographic distribution of allergists/immunologists represented by the dropped pins in Ontario, Canada as of December 2019. The inset provides a magnified view of the geographic distribution of the specialists in the urban areas

As of December 2019, there were 122 registered allergists/immunologists to serve 14.6 million residents of Ontario—the most populous province representing 38% of Canada’s population [4, 5]. However, there exists a large geographic barrier between patients who live in remote communities and specialists who predominantly practice in urban centres (Fig. 1). The shortage of the specialists, compounded by the geographic distance between patients and specialists, poses a significant barrier to access CIA care in a timely and effective manner. Telemedicine offers a unique opportunity to bridge this gap in care [6]. Herein, we aimed to characterize the use of telemedicine by allergists/immunologists in Ontario, Canada at the population and patient levels.

## Methods

We conducted a retrospective descriptive study that included all synchronous telemedicine visits provided by allergists/immunologists from January 1st, 2014 to December 31st, 2019. At the population level, data on the use of telemedicine in CIA were collected from an administrative database provided by the Ontario Telemedicine Network (OTN)—the non-profit organization funded by the Government of Ontario to provide the virtual care platform with synchronous audio–video call [7]. Available information included telemedicine visit date and location as well as health provider information and location. Patient level data were collected from the electronic medical records at our hospital in Toronto—an academic institution that provided the majority of telemedicine visits in Ontario. They included patient age and sex, postal code, diagnostic code, consult or follow-up, telemedicine visit date and location, and health provider information and location. Distance between patients’ residences and our hospital was calculated using the postal codes and Google Maps. The potential time travelled between these locations was estimated using the average speed of highway driving at 90 km/h. The study received approval from the institutional research ethics board.

## Results

**Fig. 2:**
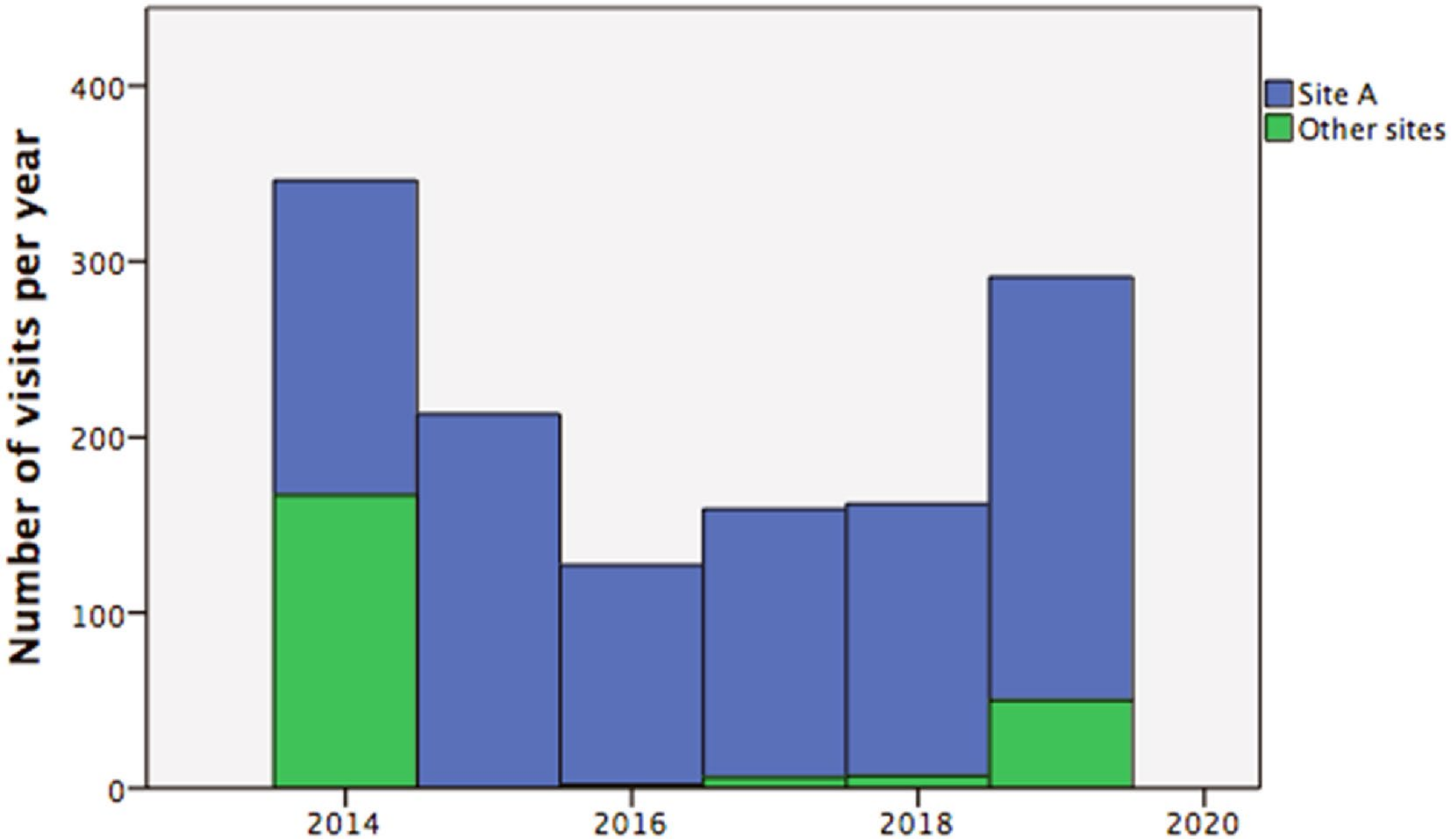
The number of telemedicine visits at the provincial level and at our hospital labeled as “Site A” from January 2014 to December 2019 in Canada

During the six-year study period, there was a total of 1298 telemedicine visits through OTN with an average of 216 visits per year (range 127–346). Only 11% of the allergists/immunologists (n = 13) used telemedicine to provide care and more than half of the visits were provided by a single physician at our hospital labelled as site A. While the number of visits has not increased much over the years, more than 80% of the visits (n = 1066) was provided by three specialists at site A (Fig. 2). At this site, a total of 865 telemedicine visits (327 new referrals and 538 follow-ups) were available for chart review during the same study period.

In the cohort from our hospital, 66% patients were female (n = 571) and the overall average age was 46 ± 16 years old. The number of telemedicine visits remained steady with an average of 170 visits per year (range 125–213). While most patients required at least 1 follow-up via telemedicine, about 18% of patients (n = 152) did not require any follow-ups and 17% of patients (n = 145) required more than 6 follow-ups via telemedicine during the study period. Most conditions assessed and followed via telemedicine were chronic diseases, including immunodeficiency (40%), asthma (13%) and urticaria (11%). Lastly, the average potential two-way travel distance avoided per visit was 718 ± 852 km and the average potential two-way travel time avoided was 6.6 ± 5.5 h (SD).

## Discussion

Our study showed that the use of telemedicine to provide CIA care in Ontario, Canada was limited but remained steady over the years before the COVID-19 pandemic. However, the annual average of telemedicine visits in our centre was comparable to another academic centre in the US (170 vs. 153, respectively) [3]. Although telemedicine use by allergists/immunologists in other countries at the population level is unknown, it was not widely adopted in Ontario—the most populous province of Canada with one third of the nation’s population [8], as over 95% of visits were provided by only 4 physicians as shown in our study. Further, most patients in our cohort had chronic diseases and required at least one follow-up via telemedicine. Compared to other studies, the reasons for consultation via telemedicine markedly differed from the ones in our centre: one centre consisted of adverse drug reaction (66%), immunodeficiency (15%) and urticaria (5%), whereas the other centre consisted of allergic rhinitis (30%), asthma (24%) and food allergy (10%) [3, 9]. Lastly, because there are very few centres that specialize in immunodeficiency care, our study showed that telemedicine allowed patients who lived in remote areas to be connected to the allergists/immunologists in urban centres and reduced the potential travel distance, similar to the reported travel distance in another study (718 km vs. 700 km, respectively) [9, 10].

Telemedicine use in CIA has been increasing in the US, particularly in adverse drug reactions and immunodeficiency, resulting in rapid turnaround time and decreased wait time [3]. It also has been associated with significant time and cost savings, as well as high patient satisfaction compared to in-person visits [9]. Despite the evidence to support telemedicine for CIA care, it had been underutilized in Canada before the pandemic. There also has been a lower ratio of allergists/immunologists to population compared to other medicine specialties in Canada, resulting in a longer wait time for an in-person visit compared to other medicine specialties [11, 12]. Timely access to allergy care is important because appropriate diagnosis and/or management of various allergic diseases improve health-related quality of life [13]. In the era of growing demand for allergists/immunologists and increasing number of patients with allergic diseases, telemedicine would be a great tool to address this supply–demand mismatch.

This is the first study to characterize the use of telemedicine to provide CIA care in Canada at both population and patient levels. There are several limitations that merit consideration. Firstly, because of the limited information in the administrative database, we were unable to assess certain clinical parameters such as patient demographics and diagnoses at the population level that were done at the patient level. Secondly, our study did not include patients from in-person visits for comparison because the study was purely descriptive. Although we could not comment on any differences in care between in-person and telemedicine visits, telemedicine has been shown to offer equal or at least non-inferior care compared to in-person visits [10, 14, 15]. Thirdly, because diagnostic codes were used to infer the reasons of assessment at each visit, we could not ascertain the accuracy of this inference and recognized that reasons for consultations may not always be the same as the diagnoses. Lastly, because patient level data were only available in one centre, the pattern of telemedicine use at our centre may not reflect its use at other centres.

In conclusion, telemedicine was not widely used by allergists/immunologists in Ontario, Canada before the COVID-19 pandemic. It could offer a unique opportunity to connect patients who live in remote communities and allergists/immunologists who practice in urban centres in Canada. Independent of the current pandemic, our study further highlights the need for more physicians to adopt and continue telemedicine use as an alternative route to deliver care when in-person visits are less ideal, as well as for healthcare agencies to support its use as a strategic priority once the pandemic is over.

## Data Availability

All data generated or analysed during this study are included in this published article.

## References

[1] Murphy RL, Bird KT (1974). Telediagnosis: a new community health resource. Am J Public Health.

[2] Portnoy JM, Elliott T (2019). Tele-allergy: here today and rapidly advancing. J Allergy Clin Immunol Pract.

[3] Phadke NA, Wolfson AR, Mancini C, Fu X, Goldstein SA, Ngo J (2019). Electronic consultations in allergy/immunology. J Allergy Clin Immunol Pract.

[4] The College of Surgeons and Physicians of Ontario (CPSO). Ontario, Canada; 2019. https://doctors.cpso.on.ca/. Accessed 15 Dec 2019.

[5] Census of the Population, 2016. Statistics Canada; 2017. https://www12.statcan.gc.ca/census-recensement/2016/dp-pd/index-eng.cfm. Accessed 15 Mar 2021.

[6] Taylor L, Waller M, Portnoy JM (2019). Telemedicine for allergy services to rural communities. J Allergy Clin Immunol Pract.

[7] Ontario Telemedicine Network. Ontario, Canada; 2021. https://otn.ca/about/. Accessed 15 Mar 2020.

[8] 2016 Census. Statistics Canada website. 2019. https://www12.statcan.gc.ca/census recensement/index-eng.cfm. Accessed 15 Dec 2019.

[9] Waibel KH, Bickel RA, Brown T (2019). Outcomes from a regional synchronous teleallergy service. J Allergy Clin Immunol Pract.

[10] Waibel KH (2016). Synchronous telehealth for outpatient allergy consultations: a 2-year regional experience. Ann Allergy Asthma Immunol.

[11] Canadian specialty profiles. Canadian Medical Association website. 2020. https://www.cma.ca/canadian-specialty-profiles. Accessed 1 Feb 2020.

[12] Neimanis I, Gaebel K, Dickson R (2017). Referral processes and wait times in primary care. Can Fam Physician.

[13] Meltzer EO (2001). Quality of life in adults and children with allergic rhinitis. J Allergy Clin Immunol.

[14] Brown W, Odenthal D (2015). The uses of telemedicine to improve asthma control. J Allergy Clin Immunol Pract.

[15] Portnoy JM, Waller M, De Lurgio S, Dinakar C (2016). Telemedicine is as effective as in-person visits for patients with asthma. Ann Allergy Asthma Immunol.

